# Hydroxyurea regulates the development and survival of B16 Melanoma Cells by upregulating MiR-7013-3p

**DOI:** 10.7150/ijms.52177

**Published:** 2021-03-03

**Authors:** Yi-Jie Huang, Yan Gao, Chang-Jiang Wang, Dong-Xu Han, Yi Zheng, Wen-Hua Wang, Hao Jiang, Bao Yuan, Jia-Bao Zhang

**Affiliations:** Department of Laboratory Animals, Jilin Provincial Key Laboratory of Animal Model, Jilin University, Changchun 130062, Jilin, P.R. China.

**Keywords:** miR-7013-3p, B16 melanoma cells, MITF, hydroxyurea (HU), pigmentation

## Abstract

miRNAs are a family of short, noncoding RNAs that are involved in many processes in melanoma cells. MITF acts as a master regulator of melanocyte function, development and survival by modulating various genes. Hydroxyurea (HU) is used to treat melanoma, and miRNA expression is altered after HU treatment in B16 melanoma cells. In this study, we screened for miRNAs that were upregulated after HU treatment and that targeted the MITF gene. We found that miR-7013-3p exhibited increased expression after HU treatment and could bind to MITF. miR-7013-3p inhibited melanin production, proliferation, and migration and promoted apoptosis in B16 melanoma cells. The results may provide more information on the roles of miR-7013-3p in B16 melanoma cells.

## Introduction

Melanin prevents the skin, eyes and brain from being damaged by ultraviolet radiation 1-3. In addition, melanin in the inner ear affects sound conduction 4. However, melanin is aberrantly regulated in human skin disorders such as vitiligo and melisma 5, 6. The pigment is produced by melanocytes through melanosomes and is transferred within melanocyte dendrites to adjacent keratinocytes 1. Melanoma, which stems from melanocytes, is a malignant cancer of the skin and has a tendency to metastasize to distant locations 7-9. This kind of skin cancer develops through abnormal hyperplasia of melanocytes and is characterized by rapid proliferation, resistance to apoptosis, unlimited replication, and significant increases in melanin content over time 10, 11. Although the incidences of many cancer types are decreasing, the melanoma incidence continues to increase; in addition, the incidence and mortality of melanoma are higher than those of other cancers of the skin 12. More than 1 million Americans currently suffer from melanoma according to current data from the American Academy of Dermatology (AAD) 13. All of this information suggests that more effective treatments for melanoma are urgently needed.

Microphthalmia-associated transcription factor (MITF) acts as a master regulator of melanocyte function, development and survival by modulating various differentiation-related, cell cycle progression-related and antiapoptotic genes and can act as an oncogene in melanoma 14-17. MITF can also activate the transcription of pigmentation-related genes, including tyrosinase (TYR), TYR-related protein 1 (Tyrp1), TYR-related protein 2 (Tyrp2), dopachrome tautomerase (DCT), and others 16, 18. MITF directly regulates the gene expression of the antiapoptotic factor B-cell lymphoma 2 (BCL2). Constitutive overexpression of BCL2 partially attenuates MITF deletion-induced apoptosis of primary melanoma cells and melanoma 13, 19, 20. MITF can regulate cell cycle progression by modulating cyclin-dependent kinase-2 (CDK2), which is important for melanoma clonogenic growth. Furthermore, MITF can regulate cell proliferation mediated by CDK2. In normal skin, cellular mesenchymal-epithelial transition factor (c-Met) is present on epithelial cells and melanocytes 21, 22. The c-Met receptor tyrosine kinase is a multifaceted regulator of growth, motility, and invasion in a number of lineages *in vivo*. c-Met is also one of the MITF target genes 22, 23. Overexpression of c-Met correlates with the invasive growth phase of melanoma cells 24, and knockdown of MITF can inhibit the invasion of melanoma cells by affecting the expression of c-met 19, 25, 26. These findings indicate that MITF is a potential target gene for melanoma therapy 15, 20, 27-29.

Hydroxyurea (HU) is a metabolic inhibitor of ribonucleotide reductase and an anticancer drug that inhibits the synthesis of DNA 30, 31. It works by scavenging free radicals of tyrosine to inhibit the production of deoxyribonucleotide, thereby reducing the production of ribonucleotide reductase 31, 32. HU has been previously reported as a treatment for melanoma 33, 34. In addition, miRNAs are a family of short, noncoding RNAs that can be cleaved by the DICER enzyme to form mature miRNAs after forming precursor RNA molecules in the nucleus. HU treatment can cause miRNA expression changes in cells 35, 36. However, there have been no relevant experiments to verify the expression levels of miRNAs in melanoma cells treated with HU.

We identified miRNAs that may bind to MITF with TargetScan software, which predicted that four miRNAs may regulate MITF gene expression. We then chose the miRNAs upregulated by HU treatment to study the roles of miRNAs in the treatment of melanoma. In this experiment, we expected to find influences of miRNAs on MITF gene expression and transcription under HU stimulation. In addition, we sought to determine whether miRNAs affect melanogenesis, proliferation, apoptosis, and migration in B16 cells by regulating MITF gene expression.

## Methods

### Materials

HU with over 98% purity was obtained from Sigma and dissolved in dimethyl sulfoxide (DMSO) to make a stock solution.

### Cell culture

Murine B16 melanoma cells were obtained from the National Infrastructure of Cell Line Resource (China) and cultured with high-glucose DMEM containing 5% heat-inactivated FBS, 100 units/ml penicillin, and 100 µg/mL streptomycin in a humidified atmosphere with 5% CO_2_ at 37 °C.

### RNA isolation and quantitative RT-PCR (qRT-PCR) detection

Total RNA was extracted by using TRIzol reagent (Invitrogen) according to the manufacturer's protocol. Next, cDNA was obtained with a FastQuant RT kit, and raw data were obtained by qRT-PCR with SuperReal PreMix (SYBR Green). GAPDH was used as an internal control for the mRNA qRT-PCR analysis, while the small nuclear U6 was used as an internal control for miRNA quantitation. The mRNA and miRNA primers used in these assays are listed in Table 1.

### Melanin content measurement

Melanin content was measured as previously described 16. Briefly, 24 h after transfection, B16 melanoma cells were collected, washed with phosphate-buffered saline (PBS) three times, dissolved in 1 mL of 1 mM NaOH and incubated at 80 °C for 30 min. The total melanin content was measured at 475 nm and normalized to the total cell number. All experiments were performed in triplicate.

### Detection of apoptosis by flow cytometry

Flow cytometry was used to detect B16 melanoma cell apoptosis in order to evaluate the effect of miR-7013-3p on cells. To analyze apoptosis, we performed an assay in strict accordance with the apoptosis assay kit instructions (Tianjin Sun Gene Biotech Co., Ltd, Tianjin, China). B16 cells were transfected with a mimic negative control (NC) and miR-7013-3p mimic for 24 h, the cells and culture medium were harvested, and the cells were digested with trypsin without EDTA. The cells were then centrifuged at 1000 r/min for 5 min and washed with ice-cold PBS; we repeated this step 3 times. Then, 5 µL of FITC solution and 5 µL of propidium iodide (PI) were added to each centrifuge tube. The cells were incubated for 15 min at room temperature in the dark. Finally, we analyzed apoptosis via flow cytometry within 2 h.

### Cell cycle assay

Cells were incubated with PI for 15 min in the dark, and then the cell cycle was detected by flow cytometry.

### miRNA mimic transfection and RNA interference

All miRNA mimics were purchased from Guangzhou RiboBio Biotech Co., Ltd. The siRNAs were synthetized by GenePharma (Shanghai, China). B16 cells were seeded at a density of 4×10^5^ cells per well in a 6-well plate. Transfection was performed with a Lipofectamine 2000 Transfection Kit (Thermo Fisher Scientific, USA) according to the manufacturer's protocol. After transfection, the cells were incubated for 24 h for gene expression analysis. The siRNA sequences are shown in Table S1.

### Wound-healing migration assay

A wound-healing assay was used to evaluate cell migration. When B16 melanoma cells grew to 80-90% confluence, 10 µL pipette tips were used to scratch a cell-free trace in the middle of the 6-well plate. After 24 h of incubation, the cell scratches were observed by optical microscopy. Finally, the average width of the scratches was calculated using Image-Pro Plus to determine the cell migration changes.

### Proliferation assay

B16 melanoma cells were transfected with the mimic NC and miR-7013-3p mimic for 24 h. Then, cells were collected for the proliferation experiment and seeded in an E-plate at a density of 5000 cells/well. Proliferation was monitored every 15 min for a minimum of 18 h by recording the cell impedance produced as the cells attached and detached from the gold electrodes in the CIM and E-plates. Real-Time Cell Analysis (RTCA) software was used to generate a survival curve and estimate the cell survival or cell index (CI). The CI correlated directly with the cell number. The data are expressed in bar graphs as the CI % relative to the control.

### Western blot analysis

Forty-eight hours after transfection, protein lysis buffer was used to lyse B16 melanoma cells. Next, the proteins were transferred to PVDF membranes for 1 h. After protein transfer, the PVDF membranes were blocked in Tris-buffered saline with Tween-20 (TBST) containing 5% bovine serum albumin (BSA) for 90 min and then incubated with rabbit polyclonal MITF IgG (ImmunoWay Biotechnology Company, Newark, DE, USA), rabbit polyclonal TYR IgG (Abcam, Cambridge, MA, USA) and rabbit polyclonal GAPDH (Cell Signaling Technology, USA) primary antibodies at 4 °C overnight before being washed 3 times with TBST for 15 min per wash. After being washed with TBST, the membrane were incubated with secondary antibodies for 55 min, after which the membranes were washed with TBST 3 times for 15 min each. The final protein bands were visualized with a chemiluminescent substrate, and protein quantification was performed with the ImageJ program.

### Statistical analysis

All statistical analyses were performed using SPSS 19.0 software (IBM, SPSS, Chicago, IL, USA). Unpaired t tests were used for two-group comparisons, and all data are expressed as the mean ± SEM from three independent experiments. Comparisons among three or more groups was performed by independent-sample tests. Statistical significance was indicated by p values less than 0.05 (*p <0.05).

## Results

### Identification of differentially expressed miRNAs that bind to MITF after HU treatment

**Figure 1:**
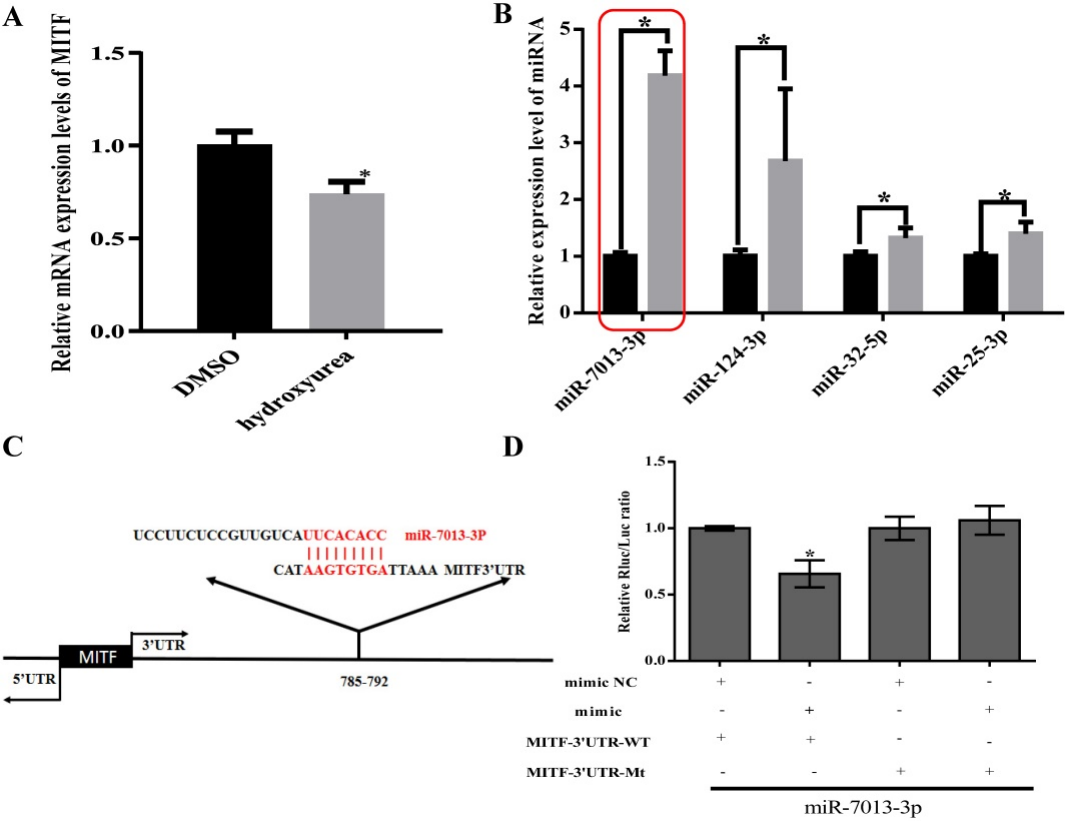
** Identification of differentially expressed miRNAs after HU treatment that bind to MITF.** After stimulation of B16 cells with HU (400 µM) for 24 h, MITF gene expression levels (A) and miRNA expression levels were altered (B). (C) Predictive maps of complementary targeting sites for miR-7013-3p and the MITF mRNA 3'UTR. (D) Luciferase activity after cotransfection with the MITF-3'UTR WT plasmid and miR-7013-3p mimic. The data summarize three independent experiments and are given as the mean±SD. *P<0.05.

We used TargetScan to predict miRNAs that bind to MITF and measured MITF gene and miRNA expression levels after stimulating B16 cells with HU (400 μM) for 24 h 36, 37. We found that MITF gene expression decreased, while miR-25-3p, miR-32-5p, miR-124-3p and miR-7013-3p expression increased (Fig. 1A-1B). We chose miR-7013-3p for further examination of the effect of this miRNA on B16 melanoma cells after treatment with HU. Information on the base complementary region between miR-7013-3p and the MITF 3'UTR was acquired via the TargetScan program (http://www.targetscan.org/) (Fig. 1C). Then, to further confirm that miR-7013-3p targets the MITF 3'UTR, we successfully mutated the target complementary sequence AAGTGTGA to TTCACACT (Table S1) and constructed an MITF 3'UTR wild-type (WT) plasmid and an Mitf-3'UTR mutated (MT) plasmid. Finally, 293T cells were cotransfected with the constructed plasmids and miR-7013-3p mimic. As we expected, cotransfection of the pmiR-Mitf-3'UTR WT plasmid and miR-7013-3p mimic into 293T cells reduced the luciferase activity (by 34%). However, cotransfection of cells with the Mitf-3'UTR MT plasmid and miR-7013-3p mimic did not reduce luciferase activity (Fig. 1D). Therefore, it can be concluded that miR-7013-3p can regulate MITF expression by directly targeting the MITF gene.

### MiR-7013-3p inhibited pigmentation

**Figure 2:**
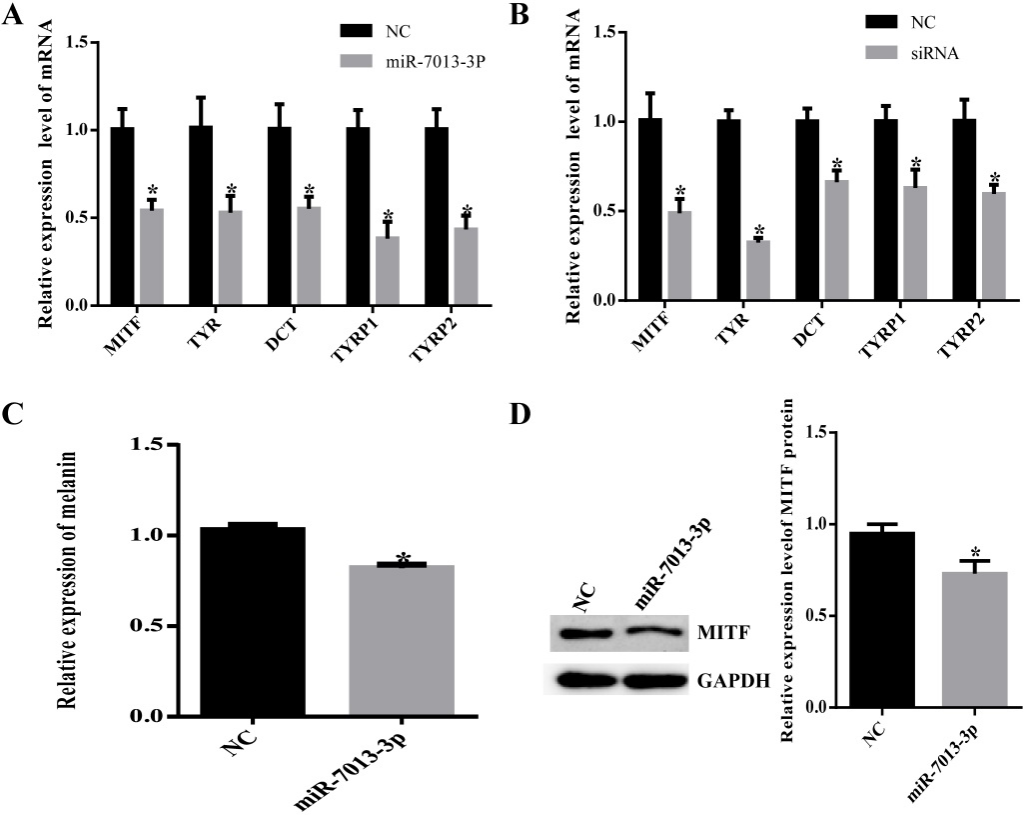
** MiR-7013-3p inhibited pigmentation.** (A) Relative MITF and pigmentation-related gene expression after transfection with the miR-7013-3p mimic. (B) Relative MITF and pigmentation-related gene expression after transfection with MITF siRNA. (C) Melanin levels in B16 melanoma cells transfected with the miR-7013-3p mimic and mimic NC. (D, E) Relative protein expression levels of MITF after transfection with the miRNA-7013-p mimic and mimic NC. The data summarize three independent experiments and are given as the mean±SD. *P<0.05.

To detect the efficiency of transfection, we transfected B16 cells with mimic NC with fluorescent markers and subsequently detected red fluorescence in the cells, indicating that the mimic NC was successfully transfected into B16 cells. (Table S2). We examined MITF and pigmentation-related gene (TYR, DCT, TYRP1, TYRP2) mRNA levels after transfection of B16 cells with the miR-7013-3p mimic for 24 h to further verify that miR-7013-3p affects MITF and pigmentation-related gene expression and regulates melanin synthesis (Fig. 2A). MITF siRNA was also transfected as a positive control into B16 cells (Fig. 2B). As expected, MITF levels and pigmentation-related genes significantly decreased after transfection (P < 0.05). In addition, the expression levels of melanin after transfection with the miR-7013-3p mimic were lower (P < 0.05) than those after transfection with the mimic NC (Fig. 2C). Moreover, Western blot analysis showed that the protein expression levels of MITF in B16 melanoma cells transfected with the miR-7013-3p mimic were lower than those in cells transfected with the mimic NC (Fig. 2D). Taken together, the results suggest that overexpression of miR-7013-3p inhibits both the mRNA and protein expression of MITF.

### MiR-7013-3p inhibited proliferation in B16 melanoma cells

**Figure 3:**
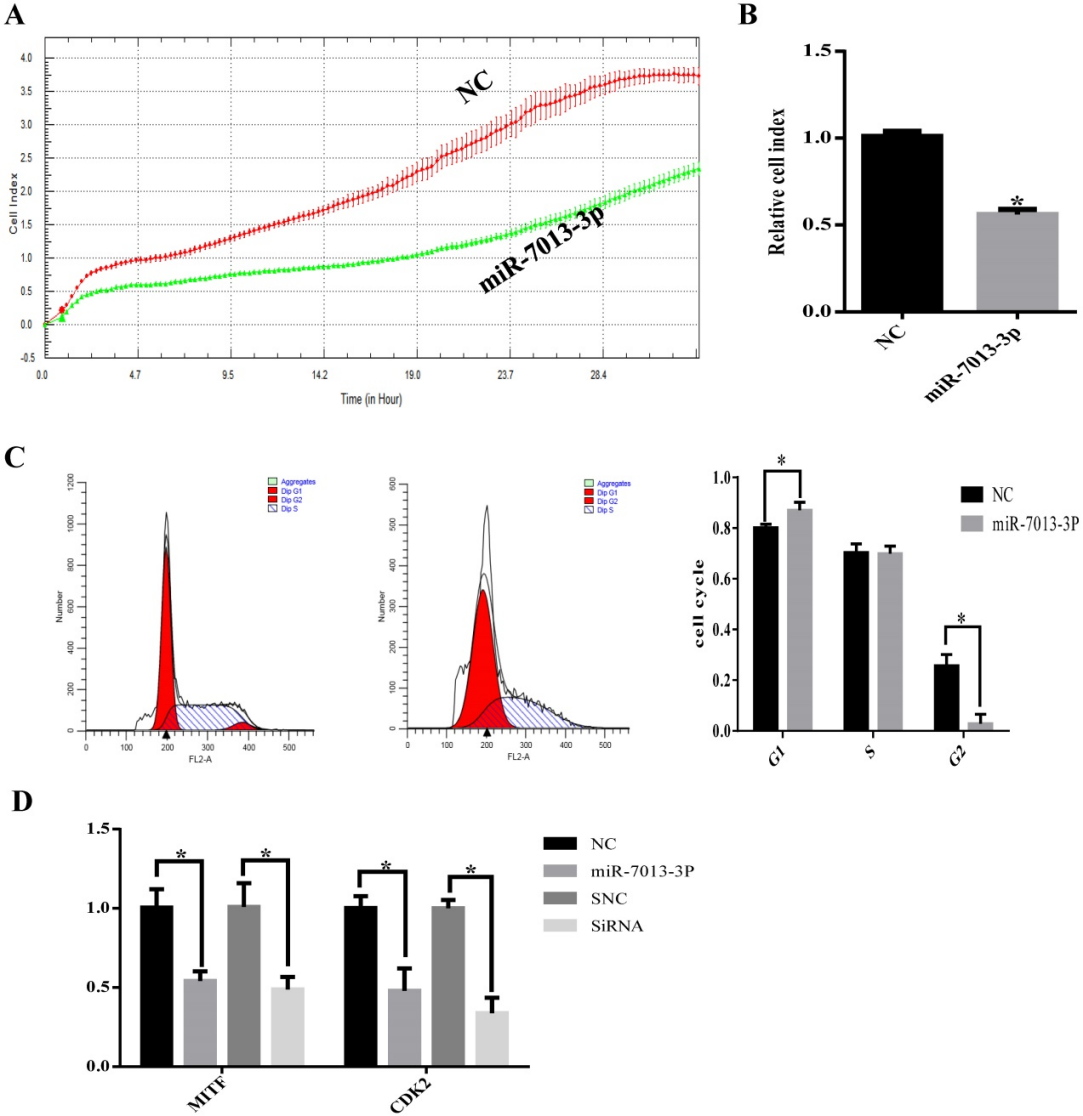
** MiR-7013-3p inhibited proliferation in B16 melanoma cells.** (A) The RTCA system was used to measure B16 melanoma cell proliferation. (B) Relative ratio of cell proliferation after transfection with the miR-7013-3p mimic compared with the mimic NC. (C) Flow cytometry was used to measure the cell cycle distribution. (D) qRT-PCR was used to analyze relative CDK2 gene expression after transfection with the miR-7013-3p mimic and MITF siRNA. The data summarize three independent experiments and are given as the mean±SD. *P<0.05.

The RTCA results showed that transfection with the miR-7013-3p mimic inhibited B16 melanoma cell proliferation (Fig. 3A). To determine the possible intrinsic mechanism by which miR-7013-3p overexpression inhibits B16 melanoma cell proliferation, we detected the cell cycle phase distribution by flow cytometry with PI staining. We found that the compared with the control treatment, overexpression of miR-7013-3p in B16 melanoma cells induced G0/G1 phase accumulation (Fig. 3B). The qRT-PCR results showed that CDK2 mRNA expression levels decreased significantly after transfection with the miR-7013-3p mimic (MITF siRNA was used as a positive control) (Fig. 3C). The results also showed that overexpression of miR-7013-3p significantly reduced CDK2 gene expression and inhibited proliferation in B16 melanoma cells.

### MiR-7013-3p promoted apoptosis in B16 melanoma cells

**Figure 4:**
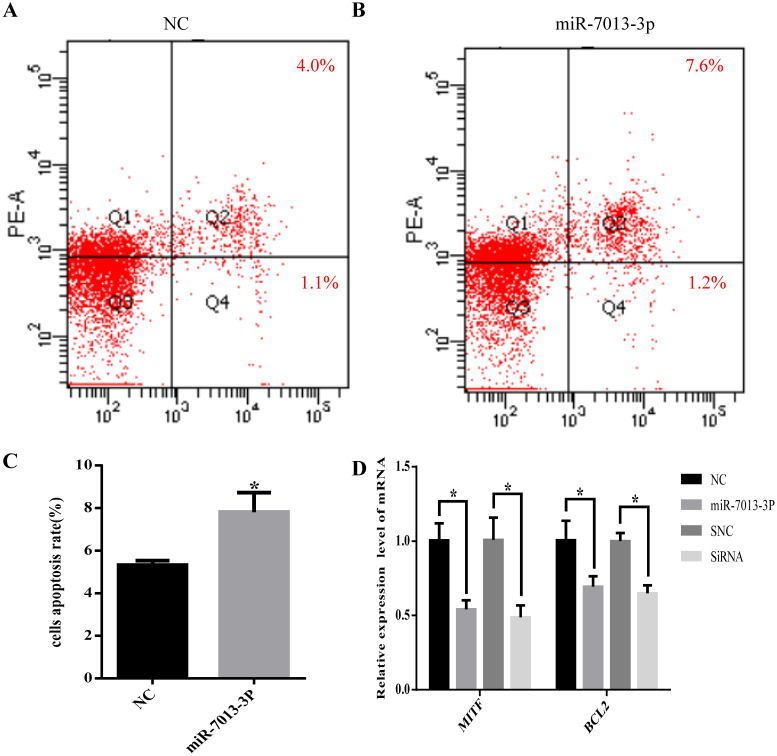
** miR-7013-3p promoted apoptosis in B16 melanoma cells.** (A-C) Flow cytometry was used to measure apoptosis. The apoptosis rate of the cells of the miR-7013-3p overexpression group (Fig. 4B) was higher than that in the NC group (Fig. 4A). (D) The qRT-PCR results show the relative expression of BCL2 genes after transfection with the miR-7013-3p mimic and MITF siRNA. The data summarize three independent experiments and are given as the mean±SD. *P<0.05.

The flow cytometry results showed that the apoptotic rate of B16 melanoma cells was significantly increased after overexpression of miR-7013-3p (P <0.05, Fig. 4A-C). In addition, the qRT-PCR results showed that the BCL2 mRNA expression level was reduced significantly after transfection with the miR-7013-3p mimic; MITF siRNA was used as a positive control (Fig. 4D).

### MiR-7013-3p inhibited cell migration

**Figure 5:**
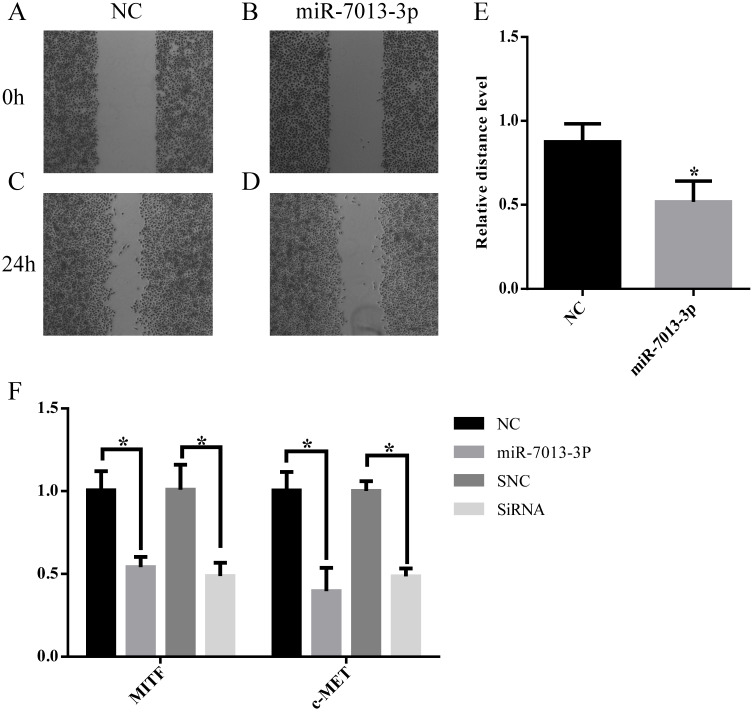
** MiR-7013-3p inhibited cell migration and invasion.** (A-E) The migration of B16 melanoma cells was measured by a wound-healing assay. (F) The qRT-PCR results show the relative expression of BCL2 genes after transfection with the miR-7013-3p mimic and MITF siRNA. The data summarize three independent experiments and are given as the mean±SD. *P<0.05.

Wound-healing migration assays were used to evaluate the migration of miR-7013-3p mimic-transfected B16 melanoma cells. As shown in Fig. 5A-5E, miR-7013-3p mimic-transfected cells showed significantly reduced migration compared with mimic NC-transfected cells. The qRT-PCR results showed that the c-MET mRNA expression level was reduced significantly after transfection with the miR-7013-3p mimic; MITF siRNA was used as a positive control (Fig. 5F).

## Discussion

Malignant melanoma is the most aggressive form of cutaneous carcinoma, with high metastatic potential and high resistance to conventional chemotherapy drugs 38, 39. The incidence of malignant melanoma has been increasing worldwide; although melanoma was a rare cancer a century ago, the average lifetime risk of melanoma is now 1 in 50 in many Western populations 39.

MITF is considered to be the master regulator of melanocytes and an oncogene in melanoma. It is essential for the proliferation and survival of melanoma cells 40. MITF stimulates melanin production by activating the transcription of pigmentation genes, including TYR, TYRP1, TYRP2 and DCT. MITF also regulates some genes that are important for melanoma survival (e.g., BCL2), proliferation (e.g., CDK2) and metastatic potential (e.g., c-MET). As an antiapoptotic factor, BCL2 can increase the antiapoptotic ability of melanoma cells. Knockdown of BCL2 can cause melanoma cells to exhibit increased apoptosis rates upon external stimulation and can improve the efficiency of drug action 41-44. CDK2 is a member of the protein kinase family. It plays important roles in regulating various events of the eukaryotic cell division cycle. Accumulating evidence suggests that overexpression of CDK2 may lead to abnormal cell cycle regulation, which may be directly related to overproliferation of cancer cells 45-48. Hepatocyte growth factor (HGF), a scatter factor and tumor cytotoxic factor, is a large multidomain heterodimeric protein that belongs to the HGF cytokine family and is the exclusive ligand of c-MET 49, 50. Preclinical studies have shown that enhancement of HGF/c-MET activity can increase the proliferation of melanoma cells 51, increase the invasive ability of melanoma cells 52, 53 and protect melanoma cells from apoptosis 22. These results indicate that MITF can be used as a target gene for melanoma treatment 15, 54, 55.

HU therapy is used for sickle cell anemia 56, transfusion-dependent β-thalassemia 57, melanoma 34 and other conditions. miRNAs that were differentially expressed under HU treatment are also involved in these treatments 35, 36, 58. These differentially expressed miRNAs regulate the expression of some genes to affect cell apoptosis, proliferation, migration and invasion. Extensive reports indicating the functions of various miRNAs in melanoma cells have been published 59. miRNAs play important roles in melanoma cells; for example, miR-137 and miR-182 can inhibit the invasion of melanoma cells through downregulation of MITF and other oncogenic target genes 25, 26. However, the function of miR-7013-3p has rarely been reported. In this study, we found that miR-7013-3p expression was upregulated after HU treatment. HU reduced MITF gene expression in B16 melanoma cells, and miR-7013-3p assisted HU in reducing MITF gene expression levels. MITF acts as a transcriptional regulator to mediate the pigmentation, proliferation, apoptosis and migration of B16 melanoma cells. Notably, MITF suppression has been found to improve the sensitivity of melanoma cells to drugs 27. In this study, miR-7013-3p was found to inhibit the function, proliferation and migration of B16 melanoma cells and to promote apoptosis of these cells by regulating the MITF gene directly. The findings of this study will help improve our understanding of the regulatory functions of miRNAs in melanoma, enrich current knowledge regarding the mechanism underlying miRNA-assisted drug function and provide a reference for later research on melanoma cells.

## Conclusion

In conclusion, our study demonstrates that HU treatment alters miRNA expression and that such miRNA changes are involved in the effects of HU on cells. For example, upregulation of miR-7013-3p inhibits melanin production, proliferation, and migration and promotes apoptosis in B16 melanoma cells. The findings add to the growing body of evidence that miRNAs are important mediators of the therapeutic effects of HU on melanoma.

## Supplementary Material

Supplementary file legends and figures.

Supplementary file.

## Figures and Tables

**Figure 6:**
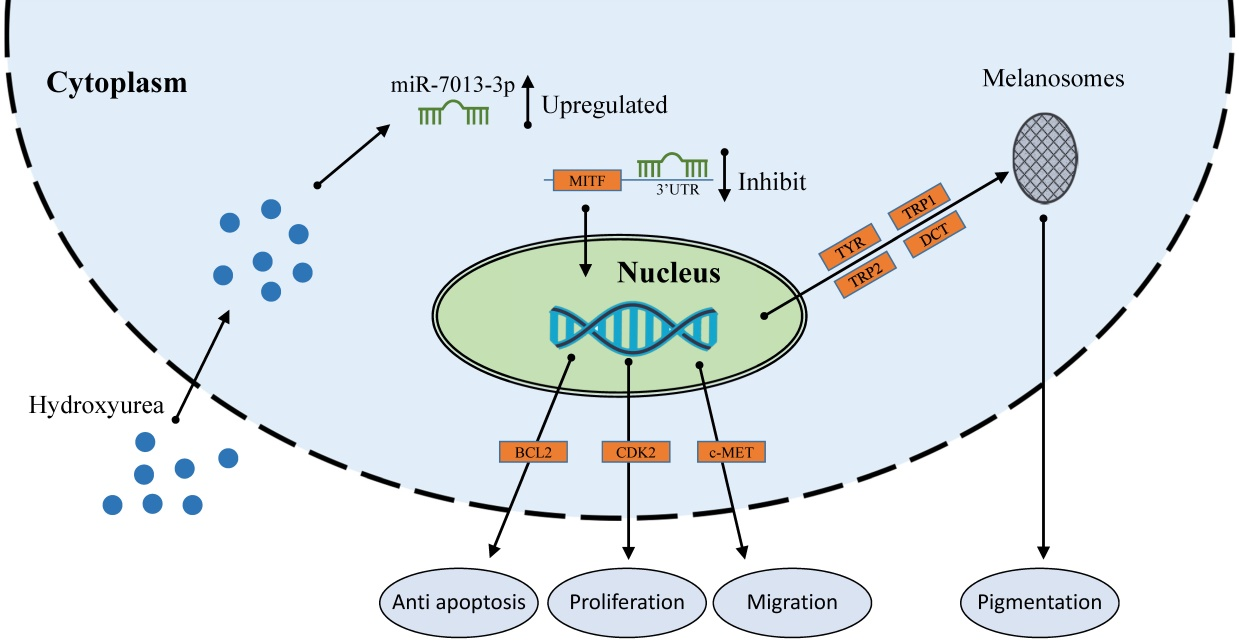
[Legend]

**Table 1:** Primers used for RT-qPCR

Primer name	Sequence (5'-3')
GAPDH F	TCATCCCTGCATCCACTGGT
GAPDH R	TGTCCCAAGTCACTGTCACAC
U6 RT	CGCTTCACGAATTTGCGTGTCAT
MITF-F	AGGACCTTGAAAACCGACAG
MITF-R	GTGGATGGGATAAGGGAAAG
DCT-F	TCTCCAGAAGTTTGACAGCCC
DCT-R	AGAGTCCAGTGTTCCGTCTG
TYRP1-F	TCGAAGCCTTCACAACCTGG
TYRP1-R	TCGTCAAAGACCGCATCAGT
TYRP2-F	TCATCTGAGCACCCCTGTCT
TYRP2-R	CGTGGAAACTGAGCCCAAAC
TYR-F	AGCCTGTGCCTCCTCTAA
TYR-R	AGGAACCTCTGCCTGAAA
miR-7013-3p-RT	CTCAACTGGTGTCGTGGAGTCGGCAATTCAGTTGAGAGGAAG
miR-7013-3p-f	ACACTCCAGCTGGGCCACACTTACTGTTGCCTCT
c-MET-F	GCATGTCAGCATCGCTCAA
c-MET-R	TGCAGGCCCAGCTGTTTC
ur	CTCAAGTGTCGTGGAGTCGGCAA
BCL2-F	CTGTGGATGACTGAGTACCT
BCL2-R	AGCCAGGAGAAATCAAACAG
miR-124-3p-RT	CTCAACTGGTGTCGTGGAGTCGGCAATTCAGTTGAGTGGCATTC
miR-25-3p-RT	CTCAACTGGTGTCGTGGAGTCGGCAATTCAGTTGAGTCAGACCG
miR-32-5p-RT	CTCAACTGGTGTCGTGGAGTCGGCAATTCAGTTGAGTGCAACTT
miR-124-3p-F	ACACTCCAGCTGGGTTAAGGCACGCGGTGA
miR-25-3p-F	ACACTCCAGCTGGGCATTGCACTTGTCTCG
miR-32-5p-F	ACACTCCAGCTGGGTATTGCACATTACTAA
CDK2-R	GGGTCCATCAAGCTGGCAGA
CDK2-F	CCACAGGGTCACCACCTCAT
