# Supplementary material for: Hydroxyurea regulates the development and survival of B16 Melanoma Cells by upregulating MiR-7013-3p
Source: Int J Med Sci. 2021 Mar 3;18(8):1877–85. doi: 10.7150/ijms.52177 (PMC7976580; doi:10.7150/ijms.52177)
Supplement: Supplementary file 1 — Supplementary file legends and figures. [file ijmsv18p1877s1.pdf]

S1 File. Construction of the pmiR-MITF-3’UTR-MUT reporter plasmid

The full-length 3’UTR of MITF mRNA was cloned between the XhoI and NotI sites of the pmiR-RB-REPORT^{TM} plasmid. To disrupt the binding site of the MITF 3’UTR, the target sequence AAGTGTGA (785-792) was mutated into TTCACACT to generate the pmiR-MITF-3’UTR-MUT plasmid. The primers used in the colony experiment were as follows:

MITF F: GCGCTCGAGCAGACAAATCTAGCAGTCATTTTCA
MITF R: AATGCGGCCGCATAAAAGTAGTCTTTCTTGGGT
MITF-MUT F: TTCCTCATTTCACACTTTAAATTTTTCATAAGGTTTTTTG
MITF-MUT R:AAATTTAAAGTGTGAAATGAGGAAAACTATAACTGTGC

The PCR colony was identified after purification of the PCR product, enzyme cleavage, purification of the cleavage product, ligation and transformation.

The plasmid was extracted from the colony, and the sequence was identified by a sequencing company. The sequencing results (Fig 1) showed that the target sequences were mutated successfully.

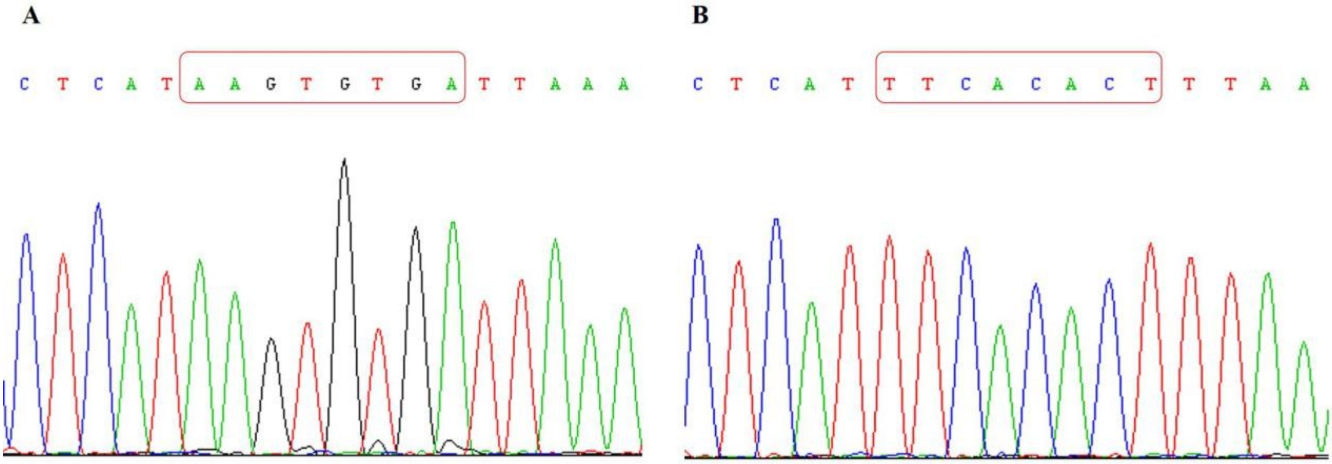

S1. Fig 1. DNA sequencing peak map. Sequence of the extracted plasmid; the target sequence AAGTGTGA (Fig 1A) was mutated into TTCACACT (Fig 1B)

S2 File. The efficiency of transfection

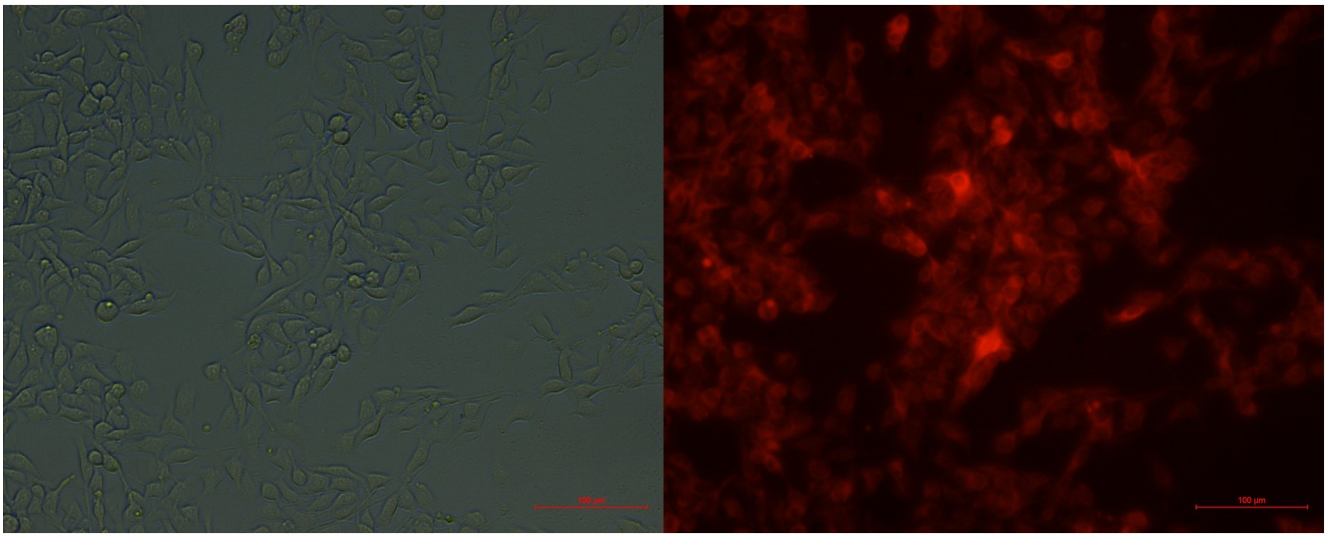

S2. File. The mimic NC with fluorescent markers was successfully transfected into B16 cells and the transfection efficiency was approximately 80-90%.
